# Strengthening simulation design and cultural safe practice: end-user evaluation of the quality simulation assurance framework

**DOI:** 10.1186/s41077-026-00454-7

**Published:** 2026-06-12

**Authors:** Melanie Barlow, Yoriko Kikkawa, Colleen Ryan, Terri Downer, Robyn Dickie, Chanchal Kurup, Kerry Reid-Searl, Aimee Lamb, Alexandra Wordworth, Natasha Eaton, Leeanne Heaton, Stephen Guinea

**Affiliations:** 1 Faculty of Health Sciences, Australian Catholic University, 1100 Nudgee Road Banyo, Brisbane, QLD Australia; 2 School of Nursing, Midwifery and Social Sciences, Central Queensland University, Rockhampton, Australia; 3 School of Health, University of the Sunshine Coast, Sippy Downs, Australia; 4 School of Nursing, Midwifery and Paramedicine, Australian Catholic University, Brisbane, Australia; 5 College of Health and Medicine, University of Tasmania, Hobart, Australia; 6 School of Nursing and Midwifery, Western Sydney University, Rydalmere, Australia; 7 Whitireia Community Polytechnic, Porirua, New Zealand; 8 Metro North Health Services, Queensland Health, Australia, Brisbane, Australia

**Keywords:** Nursing, Simulation, Higher education, Simulation-based learning, Ccurriculum, Quality, Cultural safety, Standards

## Abstract

**Background:**

Simulation-based learning is a core component of health professional education, yet designing simulations that are culturally responsive, educationally robust, and locally relevant remains challenging. Existing international standards reflect the cultural contexts in which they were developed and provide limited guidance for adapting simulation design for Australia and Aotearoa New Zealand, including effective inclusion of Australian First Nations and New Zealand Māori perspectives. The Quality Simulation Assurance Framework (QSAFe) was developed to address this gap and support culturally safe, high-quality simulation design. This study explored two research questions: (1) How do participants evaluate the applicability of the framework, including its relevance and feasibility, to their simulation practice? and (2) How does the framework support users in their design and delivery of quality simulation, including perceived helpfulness, value, and educative impact?

**Methods:**

A pilot evaluation was conducted across five tertiary institutions. Twenty-six educators used the framework to benchmark an existing simulation-based learning activity, and four participated in semi-structured interviews. Quantitative data were analysed using descriptive statistics. Qualitative data were examined using reflexive thematic analysis to explore perceptions of applicability and educative value.

**Results:**

Participants reported that the framework and supporting materials were broadly applicable to their simulation practice. Most participants rated their experience positively and indicated an intention to use the framework in future design or documentation. Four themes described the framework’s perceived value: usability, promoting best practice, supporting reflection on design, and encouraging consistent learner experience. Although the consultation and co-design element of the framework received the lowest quantitative relevance rating, findings showed this element to be least understood, particularly in relation to cultural safety and Australian First Nations and New Zealand Māori inclusion. Participants suggested improvements such as a brief orientation, video walkthroughs, and an online version with embedded guidance. Limitations included a small sample, incomplete survey responses, and challenges recruiting interview participants.

**Conclusions:**

The Quality Simulation Assurance Framework appears feasible, useful, and educative, supporting more consistent and culturally responsive simulation design. Further refinement should focus on strengthening guidance for co-design with Australian First Nations and New Zealand Māori and enhancing usability.

**Supplementary Information:**

The online version contains supplementary material available at 10.1186/s41077-026-00454-7.

## Introduction

Simulation-based learning (SBL) is a cornerstone of contemporary healthcare education, offering authentic, experiential opportunities to develop both technical, social and cognitive competencies [1]. Despite its widespread adoption, designing simulation activities that are high-quality, culturally responsive, and pedagogically sound remains a challenge, particularly for novice educators. While the Healthcare Simulation Standards of Best Practice (HSSOBP: 1) and Association for Simulated Practice in Healthcare standards (ASPiH: 2] provide robust evidence-based guidelines, their development is through a North American, or European [3] sociocultural lens. These standards have not been evaluated for sociocultural appropriateness in regions with different cultural, regulatory, health or education systems, limiting their applicability to the particular social, cultural, political, and economic contexts of Australia and New Zealand. 

The findings of Pogson, Henderson [4] strongly advocate for a structured, context‑appropriate framework. Their scoping review identified significant gaps in current approaches to evaluating the quality of simulation‑based education, noting that existing practices often rely heavily on participant feedback rather than robust frameworks capable of assessing the quality of simulation design and delivery. They highlighted three core issues: a reliance on design frameworks without adequate validation, a lack of evaluation frameworks, and the need for benchmarking practices aligned with national or international standards. Importantly, these authors concluded that the sector requires an agreed‑upon template framework to support consistent evaluation and improvement of simulation.

To address this gap, the Quality Simulation Assurance Framework (QSAFe) was developed with the aim to support simulation design that is contextually relevant and inclusive of Australian First Nations and New Zealand Māori worldviews. This paper presents findings from a pilot evaluation of QSAFe, focusing on end-user feedback (educators/academics) provided by Australian and New Zealand simulation educators regarding the tool’s utility and relevance. Specifically, the study explores two research questions:

1. How do participants evaluate the applicability (relevance, feasibility) of the new audit tool, QSAFe, to their simulation practice?
2. How does QSAFe support (helpfulness, value, educative impact) participants in the design and delivery of quality simulation?

## Background

Simulation design is a critical element of effective SBL. The Simulation Design standard of the HSSOBP encourages deliberate planning across the pre-, intra-, and post-simulation phases [5], with evidence suggesting that purposeful design enhances learner engagement, supports achievement of learning objectives, and fosters psychologically safe learning environments [6–8].

Developed using a modified Nominal Group Technique (NGT: [9, 10]) in 2023, QSAFe aims to promote best practice in simulation design tailored to the Australian and New Zealand contexts. Given the variability in higher education curricula across health disciplines, the initial focus was on undergraduate nursing programs. The modified NGT process was chosen for its ability to integrate diverse elements such as simulation standards, evolving terminology, curriculum design, accreditation requirements, and cultural perspectives. The NGT process facilitated consensus among simulation experts, resulting in a simplified and contextually grounded framework that supports novice educators in applying simulation standards effectively [11–14]. The NGT process is reported elsewhere [15] and followed a rigorous process where the facilitator adopted a neutral stance during group discussions, refraining from evaluative responses or content-based prompts. The NGT produced a clear audit trail of which preliminary findings were reviewed collaboratively to enhance transparency and credibility.

The draft framework was systematically mapped to key professional documents from both countries, including those addressing cultural considerations, undergraduate nursing program accreditation standards, national safety and quality standards, and cultural health curricula frameworks. To ensure broader relevance and methodological rigour, QSAFe mapped to both HSSOBP and ASPiH simulation standards.

### QSAFe tool and supportive resources

QSAFe was designed to both guide simulation design and benchmark existing activities to it. QSAFe comprises five key elements:

1. Simulation modalities and methodologies
2. Consultation and co-design
3. Curriculum alignment
4. Faculty preparation for delivery
5. Learner preparation for delivery

See Appendix A for an excerpt from QSAFe.

In addition to the QSAFe and recognising the need to support novice simulationists, an Education Handbook was developed offering detailed information on foundational simulation concepts referenced in QSAFe. The Handbook was co-developed with Australian First Nations and New Zealand Māori cultural advisors. This ensures the resource acknowledges Indigenous values and priorities. Cultural appropriateness and relevance across both Australian and Aotearoa New Zealand contexts, for use of language, diversity, equity, and inclusion, were considered.

Included in the Handbook is a matrix aligning QSAFe elements with Australian and Aotearoa New Zealand nursing standards of practice, and health industry quality and safety frameworks. Cultural experts also contributed to mapping QSAFe against key cultural frameworks, including the *Aboriginal and Torres Strait Islander Health Curriculum Framework* [16] and Tikanga, the traditional Māori way of life.

A user guide further supports the QSAFe tool. This user guide summarises detailed information contained in the previously described Education Handbook, which was developed prior to, and independently of, this evaluation. The guide outlines the components of QSAFe and provides rationales for their inclusion, while directing readers to the Education Handbook for more comprehensive detail. References to relevant standards and the same matrix presented in the Education Handbook are also included. QSAFe soon will have a dedicated website where resources will be freely available. Current Australian Handbooks are available via the National Library of Australia [13, 14].

To answer the research questions in this study, we asked participants, primarily educators and simulation practitioners, to use QSAFe to benchmark an existing SBL activity. Digital access to the Education Handbook and the user guide were also provided.

### Study purpose

The purpose of this study was to explore participant evaluations of the Quality Simulation Assurance Framework (QSAFe); how do participants evaluate the applicability (relevance, feasibility) of the new audit tool, QSAFe, to their simulation practice, and how does QSAFe support (helpfulness, value, educative impact) participants in the design and delivery of quality simulation?

## Methods

### Setting

Five tertiary institutions offering nursing degree programs were invited to participate: four universities in Australia and one polytechnic in Aotearoa New Zealand. Data collection occurred between April and August 2024. Ethics approval was obtained from all participating institutions, with overarching approval granted by the lead university (ACU2022-2860E).

### Participants

This study focused on simulation educators’ (end-users) evaluations of the QSAFe tool, Education Handbook, and user guide. Participant feedback was considered essential for critically appraising the content, usability, and purpose of the framework prior to its finalisation and dissemination [17]. Institutions were purposively sampled to ensure diversity in simulation experience and educational context. Participants in the study were educators/academics actively working in simulation-based education within higher education organisations and were not part of the research team or involved in QSAFe creation.

### Measures

Data collection occurred in two distinct phases.

### Phase one: evaluation of QSAFe – online

**Fig. 1:**
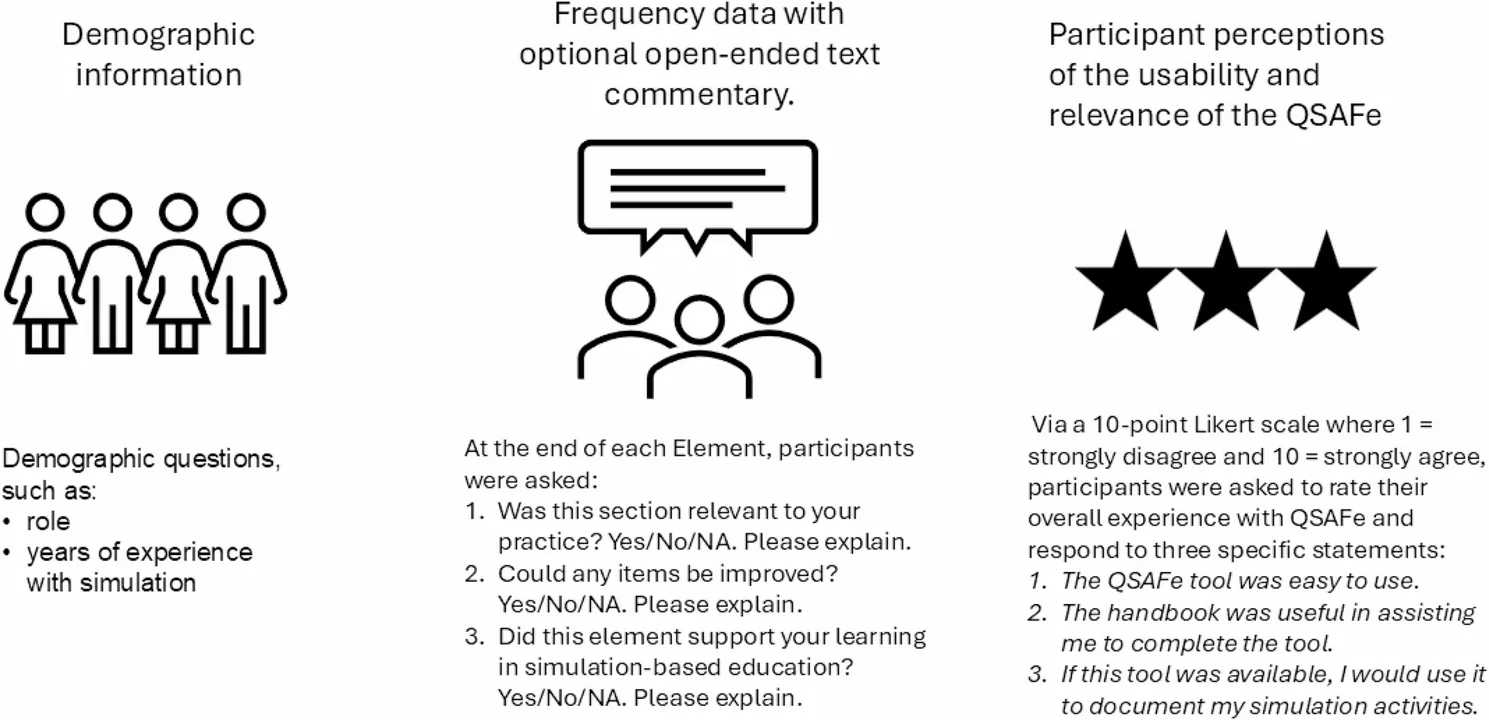
Phase one data collection

Participants were asked to use QSAFe to benchmark an existing SBL activity they had designed or currently deliver. To facilitate anonymous data collection, the QSAFe tool, the Education Handbook and the user guides were transposed into the Qualtrics^XM^ platform ([18], version 19). Anonymisation was prioritised to ensure participants felt safe sharing their experiences, with emphasis placed on evaluating the tool rather than critiquing their simulation design. This phase of data collection collected data outlined in Fig. 1.

### Phase two: evaluation of QSAFe - semi-structured interviews

Phase two of data collection comprised participants from phase one who consented to an interview via an online semi-structured interview format, see Table 1. Interviews were held by two of the research team members ensuring participants were not interviewed by individuals from their own organisation. Interviews followed an interview guide comprising questions and prompts. Interviews were held within two weeks of the participant completing the QSAFe activity (phase one).

**Table 1:** Interview questions

Focus area	Examples of interview questions
Retrospective experience of the tool	1. How user-friendly do you find the QSAFe to be? 2. Are there any features or aspects you particularly appreciate or find challenging (from content, structure, length, accessibility, formatting, voice, definitions etc.)?
Current state	3. Having now applied the tool to an existing simulation did it help identify your own gaps and strengths in simulation. 4. If so, in what way?
Future state	5. Having now been exposed to the tool, are you likely to modify your simulation design and implementation process? 6. Please provide examples of changes or improvements you’ve made as a result.

### Data analysis

Demographic and frequency data from phase one were analysed using descriptive statistics (frequencies and percentages) in Microsoft Excel^®^ exported from Qualtrics^®^. Phase two semi-structured interviews were conducted via Microsoft Teams^®^, audio recorded and transcribed. The transcription was checked for accuracy against the recording. The Principal Investigator (MB) then anonymised the transcripts prior to analysis by YK. Short answer evaluation responses and interview transcripts were qualitatively analysed using a thematic analysis approach via Braun & Clarke’s six-phase approach [19, 20] comprising familiarisation; initial coding; searching for themes; reviewing; defining/naming; reporting. Data were coded using NVIVO 14 [21] and codes developed into themes (YK). The research team sense checked and reached consensus on the codes and themes (YK). Coding was then cross-checked and codes and themes validated by SG (a nurse and simulation expert) with supporting exemplar quotes.

### Reflexivity statement

The authors acknowledge their positionality in relation to this study. Several members of the research team were involved in the development of QSAFe creating a dual role as both framework developers and evaluators and introducing the potential for positive bias in the study design, analysis, and interpretation of findings.

To mitigate potential bias, qualitative data analysis was independently conducted and overseen by YK, a non‑clinician who was not involved in the development of QSAFe and who served as an independent qualitative analysis expert. YK provided methodological oversight, supported critical interrogation of assumptions, and contributed to the rigorous examination of interpretations and emergent findings, helping to ensure balanced representation of both strengths and limitations. Cultural perspectives were provided by an Australian First Nations consultant who is a social worker; and a Māori Cultural Advisor/Tutor who provides cultural guidance and teaching within a School of Health on the North Island of Aotearoa New Zealand. The remainder of the research team comprised registered nurses and simulation experts.

## Results

Data from phases one and two were combined to respond to the research questions. Accordingly, combined data sets provided a narrative of the participant’s experience, informing recommendations for improving this version of QSAFe.

Over a five-month data collection period, research participants applied QSAFe to existing simulation activities. Twenty-six participants (*n* = 26) benchmarked activities and provided feedback within Qualtrics and via in-depth interviews (*n* = 4, av. 39 min). Interviewees were nursing academics from three different organisations across Australia who were independent to the creation of QSAFe, whose self-reported experience in simulation ranged from novice to expert.

**Table 2:** Participant demographics

Years of experience in simulation	*n*	Role* *Some participants held dual roles	*n*
0–2 years (Novice)	7	Lecturer in charge (of a unit/subject)	7
3–5 years (Junior)	3	Deputy lecturer in charge	2
6–10 years (Mid-Career)	5	Head of curriculum	1
11–20 years (Senior)	11	Facilitator/educator (of simulation activities)	12
		Simulation expert	3
		Other	1
Total	26		

**Fig. 2:**
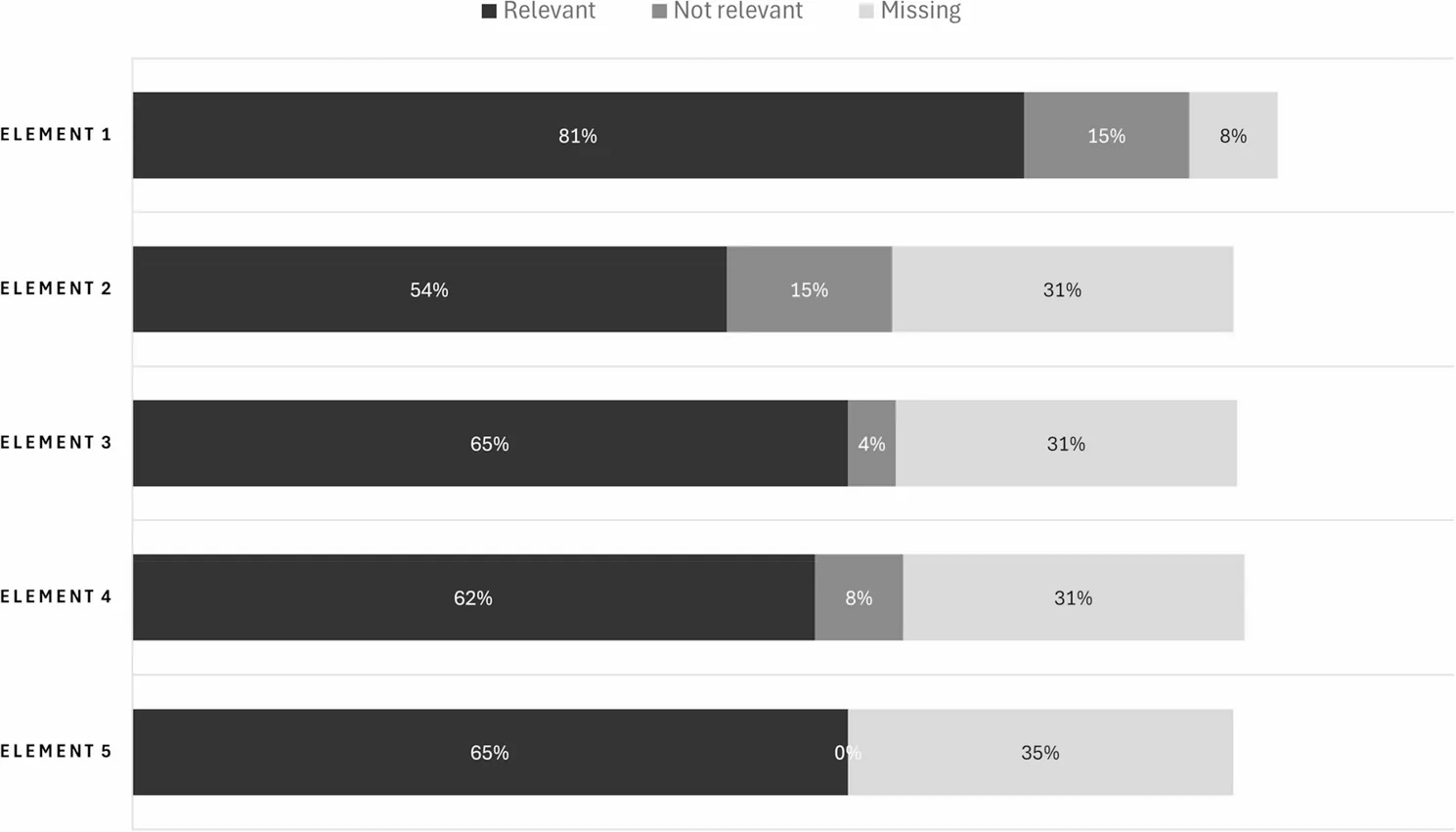
Ranked relevance of QSAFe elements from evaluation. Element 1: Modes and methods, Element 2: Consultation and co-design, Element 3: Curriculum alignment, Element 4: Faculty preparation, Element 5: Learner preparation

Overall, participants rated the contents within each QSAFe element as relevant for supporting SBL design. Figure 2 shows participants in phase one rating of the relevance of each element.

Participants’ perceptions of the usability and relevance of the QSAFe tool and its supporting educational resources were positive across both phases of feedback (Phases one and two).

### Phase one results

- Overall experience: 83% of participants (*n* = 18) rated their experience as 5 or above out of 10, demonstrating ease of use of QSAFe.
- Handbook usefulness: 89% agreed the handbook was helpful in completing using QSAFe.
- Future use: 78% indicated they would use QSAFe to document and design future simulation activities.

Inductive qualitative analysis of both phases one and two data revealed four themes and 13 subthemes related to QSAFe’s applicability and ability to support simulation design (See Table 3).

**Table 3:** Qualitative themes and sub-themes

Theme	Sub-theme
Usability	Easy to navigate Guidance for novices Fostered understanding
Promoting best practice	Consultation Design Facilitation Professional development Evaluation Debriefing
Reflecting on design	Enhancing and adapting simulation design Understanding the ‘why’ of design
Consistent student experience	Lack of consistent Quality Assurance practices Tool as a vehicle for shared understanding

### Theme 1: usability

Usability captures how easily participants at varying levels of simulation experience were able to understand, navigate, and apply the QSAFe framework. Participant responses indicated the usability of the QSAFe framework positively. Reduced usability reported was largely influenced by participants’ familiarity with simulation pedagogy (experience level). For an experienced nursing academic, the concepts were “pretty well self-explanatory” (IP3), whereas those less familiar found “the words (and) the concepts… difficult to understand” (IP4). Explanatory sections that “explained around different concepts” were “found handy” (IP1), aiding comprehension and navigation. However, both the tool and handbook were described as “quite large” with a preference for “something smaller and simple” (IP4). To improve usability, particularly for novices, participants recommended a structured onboarding, such as a brief orientation or “a video recording… [showing] ‘this is the tool… this is the purpose… how it is constructed… and how you need to use it’” (IP1). Table 4 below includes further exemplar quotes from participants.

**Table 4:**
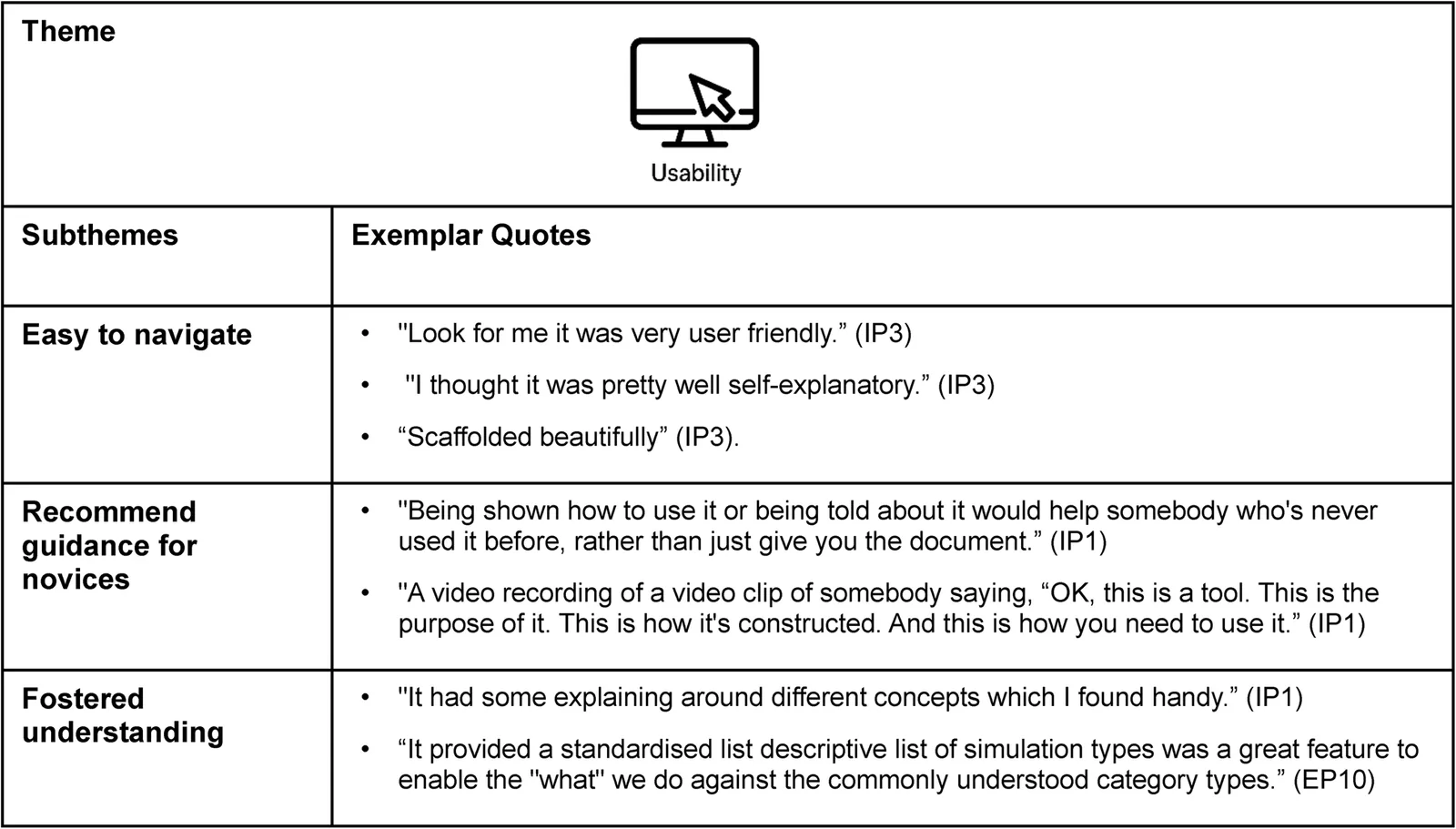
Theme 1 – usability, with exemplar quotes

*EP* Evaluation participant (phase one), *IP* Interview participant (phase two)

### Theme 2: promoting best practice

The theme Promoting Best Practice reflects how participants perceived QSAFe as a mechanism for strengthening the quality, consistency, and educational rigour of simulation design and delivery. Participants described QSAFe as offering structured guidance that supports clearer, more aligned simulation practice across individuals and teams. Refer to Table 5 for exemplar quotes for each subtheme.

**Table 5:**
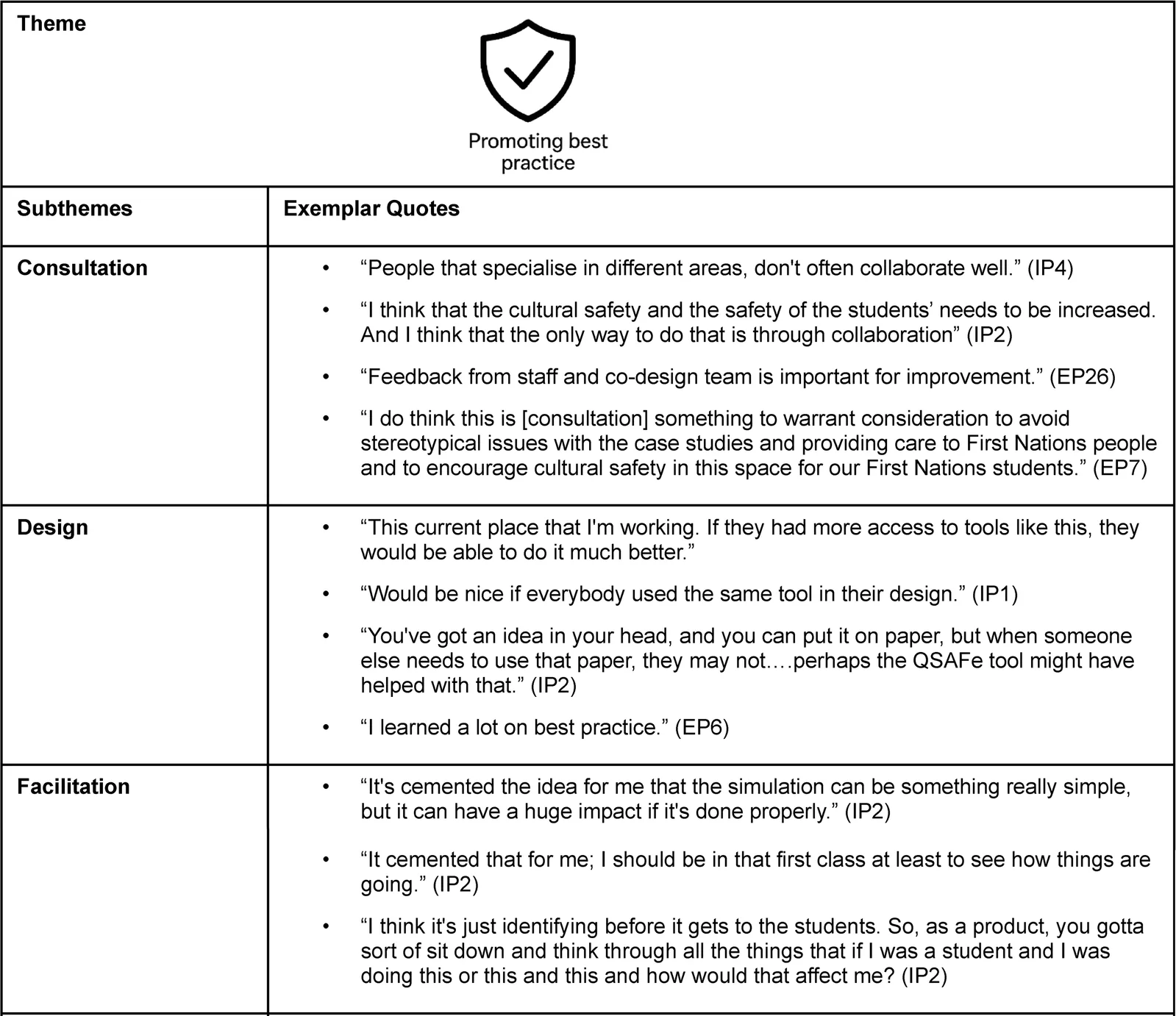

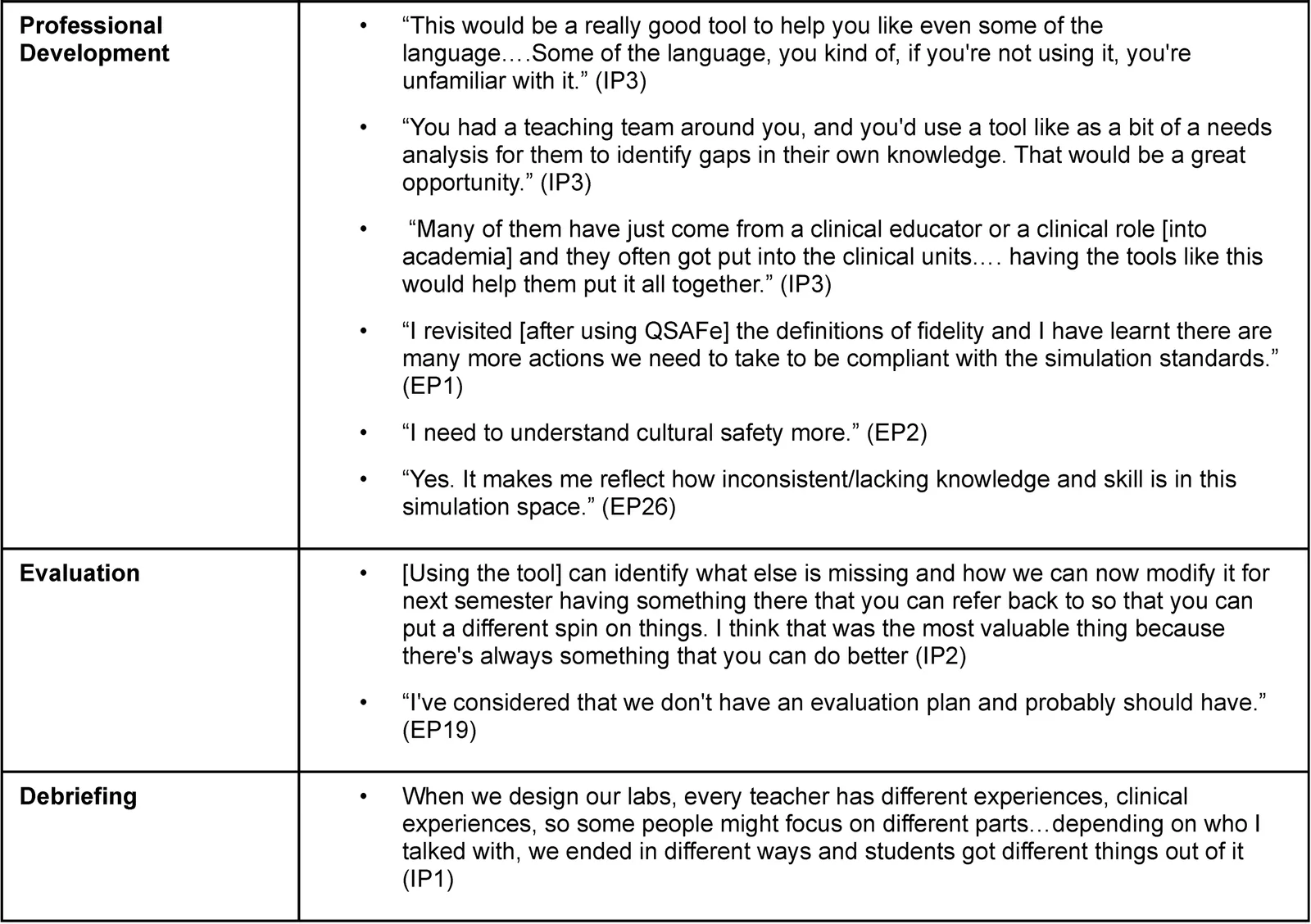
Theme 2 – promoting best practice, with exemplar quotes

*EP* Evaluation participant (phase one), *IP* Interview participant (phase two)

#### Subtheme: consultation

Even though Element 2 (Consultation and Co-design) in phase one quantitative results showed that participants found this element the least relevant (see Fig. 2), consultation emerged as a critical factor in the qualitative findings. Participants emphasised the importance of collaboration across disciplines and co-design processes to enhance cultural safety and avoid stereotypical representations in case studies. Feedback loops with staff and design teams were viewed as essential for continuous improvement.

#### Subtheme: design

Reflected participants’ recognition that access to structured tools like QSAFe supports more consistent and effective simulation design. QSAFe was seen as bridging gaps in understanding between designers and implementers and reinforcing best practice principles.

#### Subtheme: facilitation

Subtheme underscored the value of anticipating student perspectives and ensuring simulations are simple yet impactful, with checks in place before delivery.

#### Subtheme: professional development

Participants reported that QSAFe facilitated learning of simulation terminology, identification of knowledge gaps, and reflection on compliance with standards. QSAFe also prompted awareness of cultural safety and highlighted inconsistencies in simulation knowledge among staff transitioning from clinical roles to academia.

#### Subtheme: evaluation

Participants valued QSAFe for enabling reflection and iterative improvement of simulations, noting its role in identifying missing elements and prompting the development of evaluation plans.

#### Subtheme: debriefing

Participants noted that varied approaches among staff contributed to inconsistent student experiences; QSAFe prompted recognition of the need for more standardised, intentional debriefing practices.

### Theme 3: reflecting on design

This theme captures how QSAFe supported deeper reflection on the purpose, structure, and quality of simulation activities. Refer to Table 6 for exemplar quotes. Within the *subtheme Enhancing and Adapting Simulation Design*, participants described using QSAFe to update or redesign existing simulations, ensuring that activities remained responsive to emerging learner needs and evolving curricular expectations. This process encouraged educators to refine scenarios, simplify complex elements, and ensure alignment with intended learning outcomes.

The *subtheme Understanding the ‘Why’ of Design* reflected participants’ growing appreciation of the rationale behind design decisions. QSAFe prompted educators to think intentionally about each component of a simulation, fostering planning that is grounded in clear pedagogical and cultural principles rather than habit or intuition.

**Table 6:**
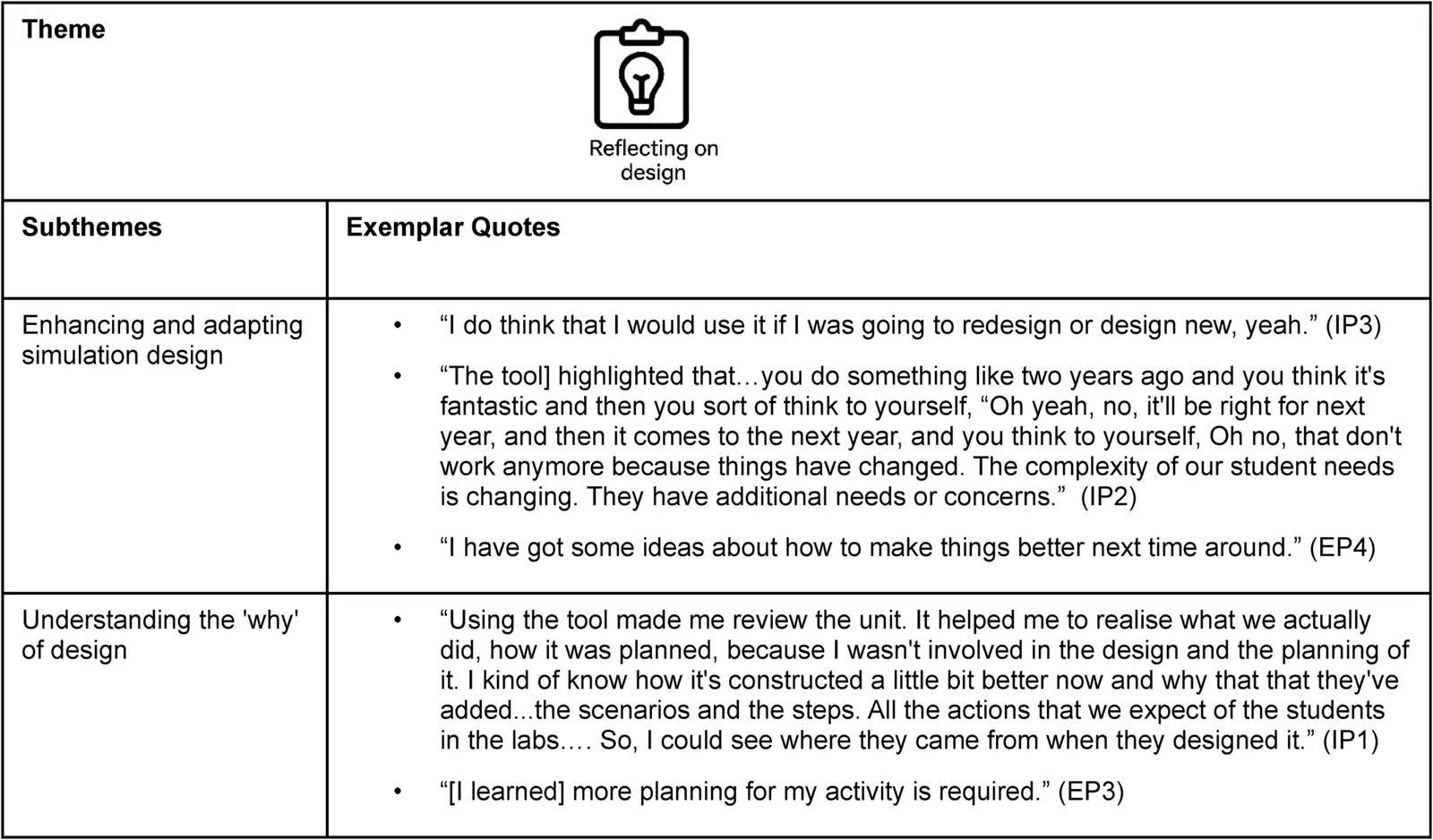
Theme 3 – reflecting on design, with exemplar quotes

*EP* Evaluation participant (phase one), *IP* Interview participant (phase two)

### Theme 4: consistent student experience

This theme reflects participants’ emphasis on the importance of delivering equitable and predictable learning experiences for students across all simulation settings. Participants highlighted in *subtheme Lack of Consistent Quality Assurance (QA) Practices*, noted that QSAFe helped reveal variability in simulation design and delivery, particularly across campuses and among casual or sessional staff. In *subtheme*,* Tool as a Vehicle for Shared Understanding*, QSAFe was seen as a unifying tool that could provide structured oversight, support shared understanding, and promote consistency, ultimately helping ensure equitable student experiences. Taken together, these findings indicate that QSAFe not only strengthens individual simulation activities but also cultivates a broader culture of reflective, high-quality practice. By encouraging educators to examine design decisions, adapt activities with purpose, and work towards greater consistency, QSAFe contributes to enhanced capability, coherence, and educational quality in simulation-based learning. See Table 7 for exemplar quotes.

**Table 7:**
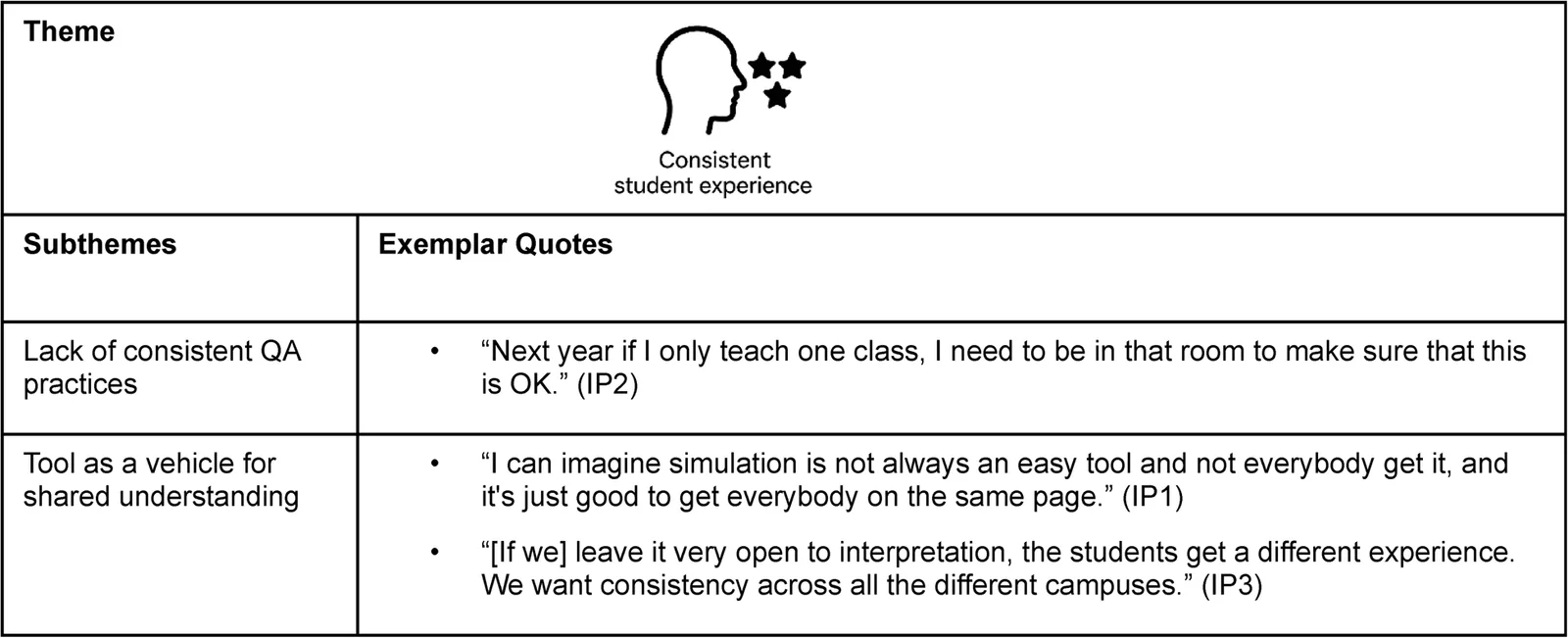
Theme 4 - consistent student experience, with exemplar quotes

*EP* Evaluation participant (phase one), *IP* Interview participant (phase two)

## Discussion

This study examined end-users’ perspectives on the applicability of the QSAFe framework to simulation practice and QSAFe’s supportive/educative impact on the design and delivery of quality simulation.

Research question one sought to understand the applicability (relevance and feasibility) of QSAFe. Participants generally judged QSAFe to be highly applicable to routine simulation practice. They emphasised the value of the tool in aiding the standardising of terminology and of simulation design processes across campuses and disciplines, thereby reducing variability in student experiences and improving alignment with curriculum outcomes and accreditation expectations. At the same time, feasibility issues emerged, most notably related to cognitive load indicated by the suggestion for a short instructional video guide as an adjunct to the Education handbook. Educators with strong backgrounds in simulation pedagogy tended to find the framework self-explanatory, whereas novice users reported difficulty with language and concepts, highlighting a need for more accessible presentation and onboarding.

Survey results positioned Consultation and Co-design as the *least relevant* element; however, qualitative data indicated it was also the least understood, particularly in relation to cultural safety and meaningful incorporation of First Nations perspectives. This divergence suggests that lower relevance ratings likely reflect limited confidence and know-how [22] rather than true lack of importance. Literature has shown that non-Indigenous practitioners frequently feel underprepared, unconfident, or insufficiently trained in cultural safety concepts, especially those requiring active partnership with diverse cultural communities [23, 24].

Participants proposed scaffolded guidance for novice educators would be helpful when first engaging with QSAFe. Several respondents also noted that the Qualtrics format used for the pilot version of QSAFe, and displayed all content at once, likely amplified perceived complexity of the framework. An interactive version of QSAFe was recommended by participants to improve usability and feasibility.

Research question two sought to understand the level of support (helpfulness, value, educative impact) QSAFe provided. Participants described QSAFe as a resource that serves multiple purposes:

1. Design scaffolding and alignment. QSAFe provided clear prompts and shared language to plan scenarios, link activities to learning outcomes, and contextualise international standards within local program structures. Notably, participants reported that the framework helped them understand the “why” behind inherited simulation designs, improving continuity and coherence when activities were designed by others. A key learning for participants regarding simulation design was the need for collaboration and consultation to meet alignment to standards, curriculum requirements, enhance the student experience, ensure sufficient resourcing and authentically represent aspects of clinical and cultural care.
2. Professional development and capacity building. Educators used QSAFe for self-assessment, peer-based needs analysis, and, in some instances, formal audit. These uses were experienced as non-threatening and supportive, enabling individuals and teaching teams to identify knowledge/practice gaps (including cultural safety) and to plan targeted development. This dual function, design tool and professional development mechanism, supports consistent practice across diverse and casualised teaching teams. As previously highlighted by [24] and Hardy, Filipenko [24], non-Indigenous practitioners need guidance and support to build confidence and competence to meaningfully consult and create active partnerships with First Nations communities.
3. Shared understanding and preparation for facilitation. The framework was viewed as a vehicle for consistency in facilitation and debriefing expectations, reducing variation between educators/classes and enhancing reliability of learner experiences across sites. Here is where QSAFe extends existing simulation standards, as recommended by Walshe, Condon [25], by incorporating strategies for culturally safe practices in simulation. This is the key difference between QSAFe and simulation standards. That is, contextual capacity building of educators to deliver quality, culturally appropriate learning and prevent unintentional harm [26].
4. Quality assurance

Participants believed usability could be enhanced further by including brief orientation sessions and short video walk-throughs to demonstrate purpose, structure, and use. A video walkthrough of QSAFe would function as a worked example and expert‑led scaffolding of information, reducing extraneous cognitive load [27], lowering any ambiguity or potential frustrations in learning how to use the tool. Guided practice has shown to support learners by minimising ambiguity and frustration [28].

### Integrating the findings

QSAFe directly addresses the gaps identified by Pogson, Henderson [4], who reported a lack of validated, structured frameworks for evaluating the quality of simulation-based education and highlighted an over-reliance on satisfaction-based evaluation rather than robust design-focused approaches. As a culturally informed quality-assurance and design tool tailored for Australia and Aotearoa New Zealand, QSAFe responds to this need by offering a consistent, transparent, and contextually grounded mechanism for strengthening simulation design processes. Findings from this study indicate that QSAFe is both relevant and feasible for routine use, with usability further enhanced through improved accessibility features such as web-based delivery, concise definitions, and orientation resources. Participants also described QSAFe as educative, supporting capacity building and reflective practice, particularly in areas where confidence tends to be lower, such as consultation, co-design, and culturally safe practice. By localising international standards and explicitly linking simulation design decisions to learner outcomes and accreditation expectations, QSAFe helps bridge the policy–practice gap and makes high-quality simulation design more actionable across programs and disciplines.

This aligns closely with the commentary by Bohnert and Lewis [3] which emphasises the broader international effort to classify competent simulation practice and the persistent challenges in achieving consistent implementation and assessment of standards. Their call for structured, competency‑focused guidance reinforces the value of QSAFe as a practical, context‑specific framework that operationalises high‑level professional standards into clear, actionable processes. In doing so, QSAFe strengthens consistency, quality assurance, and culturally grounded simulation design across diverse educational settings.

### Next steps: there is still work to be done

While Australian First Nations and New Zealand Māori experts contributed valuable guidance to the development of QSAFe, concerns remain regarding whether the perspectives currently represented, adequately reflect the diversity within and across these communities, particularly the many distinct Nations across Australia and the treaty cultural requirements of Aotearoa New Zealand. Voices from multiple Australian First Nations communities, Torres Strait Islander peoples, and New Zealand Māori communities are missing, and their input is critical for authentic cultural inclusion. This highlights an important area for future research. The next phase of work will focus on strengthening guidance for consultation and co-design in the development of culturally appropriate and culturally safe simulation activities. This research will employ a co-design methodology in partnership with First Nations Peoples across Australia and Māori communities, with the aim of documenting a stepwise co-design process that can serve as an exemplar for others engaging in similar work.

Additionally, three Australian universities are currently undertaking a study to further evaluate QSAFe by benchmarking their simulation-based curricula.

### Limitations

This study is limited by its sample size, which reduces the generalisability of findings and the strength of inferences about QSAFe’s impact. A further constraint relates to partial completion of the benchmarking task: a subset of participants (*n* = 8) provided feedback only for Element 1. Several factors likely contributed: (i) the anonymised survey platform did not allow pause–resume, discouraging completion; (ii) some users realised mid‑process they required additional resources (e.g., course outlines, simulation documentation) and chose to restart or withdraw; and (iii) for participants less familiar with simulation pedagogy, the cognitive load of evaluating complex design elements may have led to non‑completion.

Interview recruitment was also challenging, consistent with the high workload and competing priorities of simulation educators/academics. In addition, a small number of potential participants may have perceived the interviews as evaluative of their practice rather than the framework, introducing discomfort or reluctance to engage. Cultural experts in both countries were time limited. Yet this pilot provided invaluable direction in this development phase. A distinct and independent project is planned for New Zealand.

## Conclusion

The QSAFe framework offers simulation educators a structured, evidence-informed set of cues and prompts to guide the design, delivery, and evaluation of simulation-based learning. Participant feedback in this study highlighted both the inherent complexity of simulation practice and the value of QSAFe in navigating these challenges. Participants reported that QSAFe helped identify gaps in simulation design, particularly in areas such as Australian First Nations co-design and cultural safety, while also providing a coherent structure that supports reflective practice and promotes consistency across simulation activities.

Importantly, while QSAFe contributes to creating more culturally safe learning environments by prompting educators to consider culturally responsive design elements, the framework does not replace the need for ongoing individual development. Building genuine cultural awareness, capability, and responsiveness remains a critical responsibility of educators themselves. QSAFe can guide and support this work, but culturally safe practice ultimately depends on educators’ sustained engagement with cultural learning and partnership with cultural representatives.

The integration of Australian and New Zealand simulation educator end-user feedback in this evaluation will inform ongoing refinement of QSAFe and its accompanying resources, including improved accessibility, onboarding supports, and expanded guidance for consultation and co-design. As simulation-based education continues to evolve across health disciplines, QSAFe provides a practical and adaptable mechanism to strengthen quality, enhance cultural responsiveness, and translate best practice standards into actionable design processes.

## Supplementary Information

Supplementary Material 1.

## Data Availability

The datasets used and/or analysed during the current study are available from the corresponding author on reasonable request.

## Abbreviations

ASPiH: Association for Simulated Practice in Healthcare
EP: Evaluation participant
HSSOBP: Healthcare Simulation Standards of Best Practice
IP: Interview participant
QSAFe: Quality Simulation Assurance Framework
